# Strong phylogenetic inertia on genome size and transposable element content among 26 species of flies

**DOI:** 10.1098/rsbl.2016.0407

**Published:** 2016-08

**Authors:** Camille Sessegolo, Nelly Burlet, Annabelle Haudry

**Affiliations:** Laboratoire de Biométrie et Biologie Evolutive, Université de Lyon, Université Claude Bernard Lyon 1, CNRS, UMR5558, 69100 Villeurbanne, France

**Keywords:** genome size, phylogenetic inertia, transposable elements, flies

## Abstract

While the evolutionary mechanisms driving eukaryote genome size evolution are still debated, repeated element content appears to be crucial. Here, we reconstructed the phylogeny and identified repeats in the genome of 26 *Drosophila* exhibiting a twofold variation in genome size*.* The content in transposable elements (TEs) is highly correlated to genome size evolution among these closely related species. We detected a strong phylogenetic signal on the evolution of both genome size and TE content, and a genome contraction in the *Drosophila melanogaster* subgroup.

## 1. Introduction

One striking outcome of genome evolution is illustrated by the dramatic 200 000-fold variation in genome size across eukaryotes. Generally, eukaryote genome size reflects the genomic content in repeated sequences, especially in transposable elements (TEs, [1,2]). Although McClintock described TEs in the 1950s [3], it was only with the first whole genome sequencing projects that the community realized the extent of the repeatome (more than 45% of the human genome). Except for some rare cases of beneficial domestication events, TEs are seen as selfish parasitic elements that are mainly neutral or deleterious for their host [4].

Lynch and Conery proposed that genome size results from non-adaptive forces such as genetic drift and mutation. Their model predicts an accumulation of TEs—and therefore larger genomes—in species of small effective population size *N*_e_ [5]. In such species, selection to remove TEs may not be efficient compared with drift. A very intense debate on the relative role of *N*_e_ on genome size evolution among distantly related taxa followed [6–10], *inter alia* because the original model was not robust to phylogenetic control [8]. Indeed, a greater resemblance in inherited traits is expected among closely related species compared with distant ones, independently of selection or drift on these traits. Recently, a few studies focused on closely related species (reducing the number of potential confounding factors) to test for an accumulation of TEs in the genome of species with an expected reduced *N*_e_ followed by recent life-history trait changes, and reported contrasting results [11–15].

To date, quantifying the importance of phylogenetic inertia in TE content distribution remains a key question as the dynamic of TE accumulation is still poorly understood. Here, we analysed the evolution of genome size and genomic TE content in 26 *Drosophila*, using a phylogenetic framework. We estimated genomic TE content using a de novo TE assembly approach, tested the correlation between TE content and genome size among closely related species and finally estimated the phylogenetic inertia.

## 2. Material and methods

### (a) Genome size

Genome sizes were estimated using flow cytometry on 24 species. DNA content estimates were collected from the Animal Genome Size database for 23 species (http://www.genomesize.com). Cytometry measures for *D. suzukii* were performed in the laboratory on fresh samples of 4 day old females, with 10 replicates, from an isofemale line collected in France (P. Gibert).

### (b) Sequencing data

Public datasets of short read sequences were downloaded from the Search Read Archive (SRA) database, except for *D. yakuba* (provided by K. Thornton). Runs were selected with paired-end data when possible (all except *D. santomea*), sequenced from females. Run identification numbers and more details about the data are provided in the electronic supplementary material.

### (c) Repeat content

The genomic content in repeated elements was estimated for each species from de novo assembly and annotation using dnaPipeTE [16]. We filtered raw reads using unsupervised quality trimming [17] and used a random sample corresponding to 0.25× coverage. Simple repeats, satellites and low complexity elements were pooled in the ‘simple repeats' category. To test the effect of datasets' heterogeneity on TE content estimates—as clustering efficiency might vary according to read length—we simulated 0.25× datasets of varying length (40–120 bp) from the reference genome of *D. melanogaster* using ART [18].

### (d) Phylogeny reconstruction

Cytochrome *c* oxidase subunit I (*coI*) sequences were recovered for each of the 26 species using the following methodology. Homologous sequences to the *D. melanogaster* reference protein (Uniprot P00399) were identified using ncbi-tblastn. A consensus was built from the 10 best hits to account for intraspecific diversity. In parallel, sequences homologous to the reference *coI* sequence were identified by blast among dnaPipeTE contigs (mitochondrion is identified as repeated element owing to its higher coverage compared with nuclear genome). A consensus sequence was finally built between the two sequences. We obtained a 1536 bp long alignment, in respect of the protein sequences. A similar methodology was used to recover fill mitochondria sequences. Best-fit model of nucleotide substitution was selected using jModelTest v. 2.1.10 [19]. According to Bayes information criteria (BIC), a GTR + I + G model was used to reconstruct the species phylogeny by maximum-likelihood (100 bootstraps) using PhyML [20].

### (e) Phylogenetic analyses

Comparative analyses were performed using ape [21], nlme [22] and phytools [23] packages in R. Ancestral trait reconstruction of genome size was calculated using phylogenetic independent contrasts. We tested the phylogenetic signal using Pagel's *λ* [24]. Best-fitting model to the trait evolution and its covariance structure was tested among (i) absence of phylogenetic signal, (ii) neutral Brownian motion and (iii) constrained evolution Ornstein–Uhlenbeck (OU) models using generalized least squares (GLS) and selected according to minimum Akaike information criterion (AIC). We then estimated OU model parameters by maximum-likelihood.

## 3. Results

**Figure 1.**
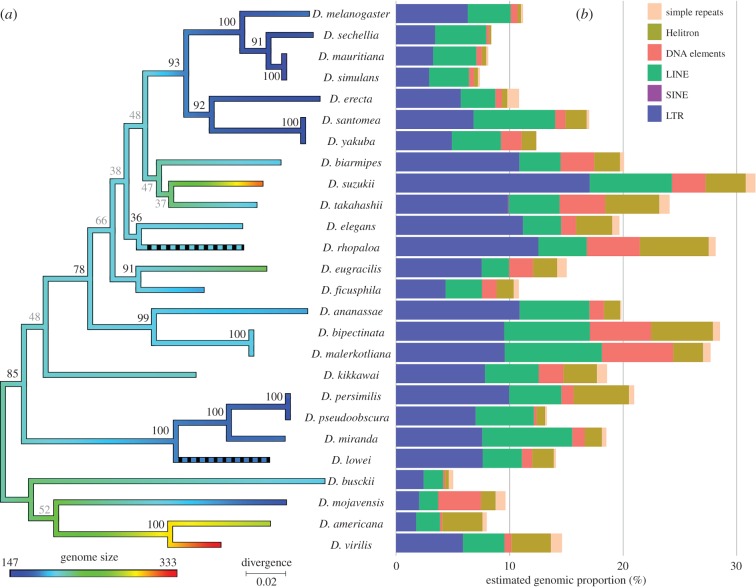
Phylogenetic tree representing genome size evolution for 26 *Drosophila* species (*a*) and their genomic content in repeated elements (*b*). Bootstrap support of each node is specified on the tree (values <70 in grey indicate less robust nodes). Colours of the branches represent genome size estimates (black dashed branches are used for lineages with unknown genome size).

We analysed genome size and TE content evolution among 26 flies. Overall, a twofold variation in genome size was detected, ranging from 147 (*D. mauritiana* and *D. simulans*) to 333 Mb (*D. virilis*). The smallest genomes (less than 180 Mb) essentially clustered into the *melanogaster* (*n* = 7) and the *pseudoobscura* (*n* = 3) subgroups (figure 1*a*). After trimming, average read length varied from 47 (*D. persimilis*) to 121 bp (*D. ficusphila* and *D. kikkawai*).

The genomic content of repeats ranged from 4.65% in *D. busckii* to 30.80% in *D. suzukii* (figure 1*b*). TEs are major components of the repeatome, essentially with LTR and LINE elements, compared with simple repeats (less than or equal to 1%). Some species exhibit a large proportion of DNA elements (6.3% in *D. malerkotliana*) and Helitron (6% in *D. rhopaloa*). Global TE content is significantly correlated with the genome size (Spearman's *ρ* = 0.43, *p* = 0.04).

We detected a significant effect of read length on the estimated TE content using simulated *D. melanogaster* data (*χ*^2^ = 1780, d.f. = 16, *p* < 2.2 × 10^−16^). The repeatome tends to be underestimated using reads shorter than 80 bp (electronic supplementary material). Removing five species with reads less than 80 bp did not affect the correlation coefficient between genome size and TE content, but the relationship became non-significant as a result of the reduced test's power (*ρ* = 0.44, *p* = 0.06).

We reconstructed the phylogeny from *coI* (figure 1*a*): 15 out of 23 nodes are robust (bootstrap values more than 70) and congruent with a phylogeny reconstructed from the full mitochondria sequences (electronic supplementary material) and with previous studies, except for two branches (*D. eugracilis* and *D. kikkawai*). The clade ancestral genome size was much larger than *D. melanogaster*'s, whose subgroup ancestor had a serious genome compaction. The phylogeny fully explains both genome size and TE content variation among the 26 flies (*λ* = 0.98, *p* = 1.45 × 10^−4^ and *λ* = 0.88, *p* = 2.19 × 10^−3^, respectively). Strong phylogenetic signal is confirmed by GLS analysis: the OU model (AIC = 262) better fits the genome size evolution than the non-phylogenetic (AIC = 270) or the Brownian (AIC = 328) model. Similar results were found for TE content (AIC of 174, 177 and 280, respectively). We detected a significantly different optimal genome size for the *melanogaster* subgroup (deviance = 257.7, *p* = 0.03).

## 4. Discussion

The evolution of eukaryote genome size remains mysterious. While the respective roles of neutral and selective forces are debated [6–10], TE accumulation emerges as a major factor of genome size variation. In this study, our estimates of the genomic TE content in 26 *Drosophila* support this claim among closely related species. We detected a strong phylogenetic signal on the evolution of both genome size and TE content, and genome contraction in the *D. melanogaster* subgroup.

So far, detailed analyses of genomic content in TEs have been restricted to model-species, because specific amplification methods (targeting one type of TE at a time) are time-consuming and fairly expensive, and whole-genome sequencing methods met technical limitations owing to the challenging assembly of repeat-rich regions. New methods allow this obstacle to be overcome by using the repeated nature of TEs to perform de novo identification from raw reads. Here, we detected a greater proportion of TEs in the genomes than previous estimations done on flies by means of genome assemblies (in which TE-rich regions are underrepresented owing to assembly difficulties). However, our estimates of TE content are congruent with previous ones ([25], *R*^2^ = 0.64, *p* = 0.04, *n* = 10). Although their genomic content remains limited in flies (15.8% on average) compared with other eukaryotes [26], TEs appear to be driving genome size in flies, like in plants [2] or in eukaryotes [1].

The best-fit OU model suggests some stabilizing selection on genome size evolution, with a significantly different optimal genome size for the *melanogaster* subgroup. The model detects the apparent genome contraction in this subgroup. While this result holds without *D. kikkawai* and *D. eugracilis* for which the position in the reconstructed phylogeny was not consistent with [27], it has to be considered with caution because a Pagel's modification of the basic Brownian model had a similar fit to the OU. It is necessary to test the constrained versus neutral evolution of the genome size on a more phylogenetically balanced sample to conclude on this point. The phylogeny fully explains the distribution of both genome size and TE content among the sampled species, while a previous study indicated that genome size varied with some life history traits (development time, body size and sperm length) in this genus (with reference to Gregory and Johnston [28]). Similarly, a very strong phylogenetic signal was found on genome size variation in liverworts [29] and evening primroses [13], independently of expected variation in *N*_e_. In those species, variation in *N*_e_ was expected as a result of changes in some life-history trait, as determining long-term *N*_e_ is very challenging and requires, for example, polymorphism estimates. Although there is evidence of some life-history traits promoting the accumulation of TEs (e.g. mating system in *Daphnia* [30] or parasitism in *Amanita* fungi [12]) owing to their impact on *N*_e_, empirical studies of specific clades accounting for phylogenetic signal are not unanimous.

Here, we have performed, we believe, the first phylogenetic analysis of genome size and genomic repeated content in a large set of *Drosophila* species. Our results suggest that the effect of life-history changes (and resulting variations of *N*_e_) on TE spread may not be detected in a short evolutionary scale owing to the major role of phylogenetic inertia. To further test the role of drift in this clade, exhaustive estimates of *N*_e_ and unbiased sampling of the phylogeny are now required.

## Supplementary Material

Datasets details

## Supplementary Material

Estimation of TE content in the reference genome of D. melanogaster from simulated datasets of different read length.

## Supplementary Material

Mitochondria phylogenetic tree

## Data accessibility

Phylogenetic data, including alignments: TreeBASE accession number 19296. (http://purl.org/phylo/treebase/phylows/study/TB2:S19296).
