# Supplementary figures and images for: Strong phylogenetic inertia on genome size and transposable element content among 26 species of flies
Source: Biol Lett. 2016 Aug;12(8):20160407. doi: 10.1098/rsbl.2016.0407 (PMC5014035; doi:10.1098/rsbl.2016.0407)

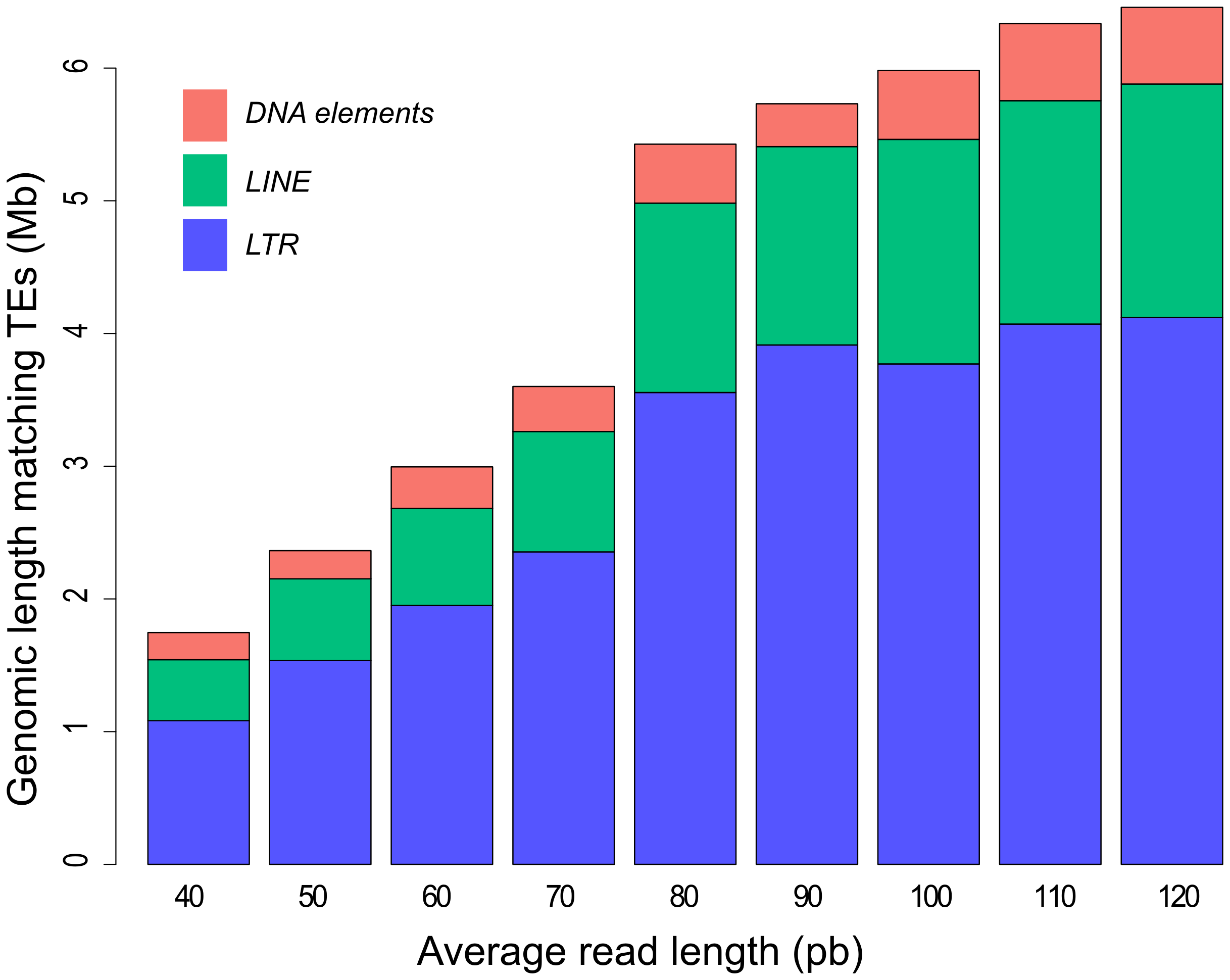

Supplement: Estimation of TE content in the reference genome of D. melanogaster from simulated datasets of different read length. [file rsbl20160407supp2.png]

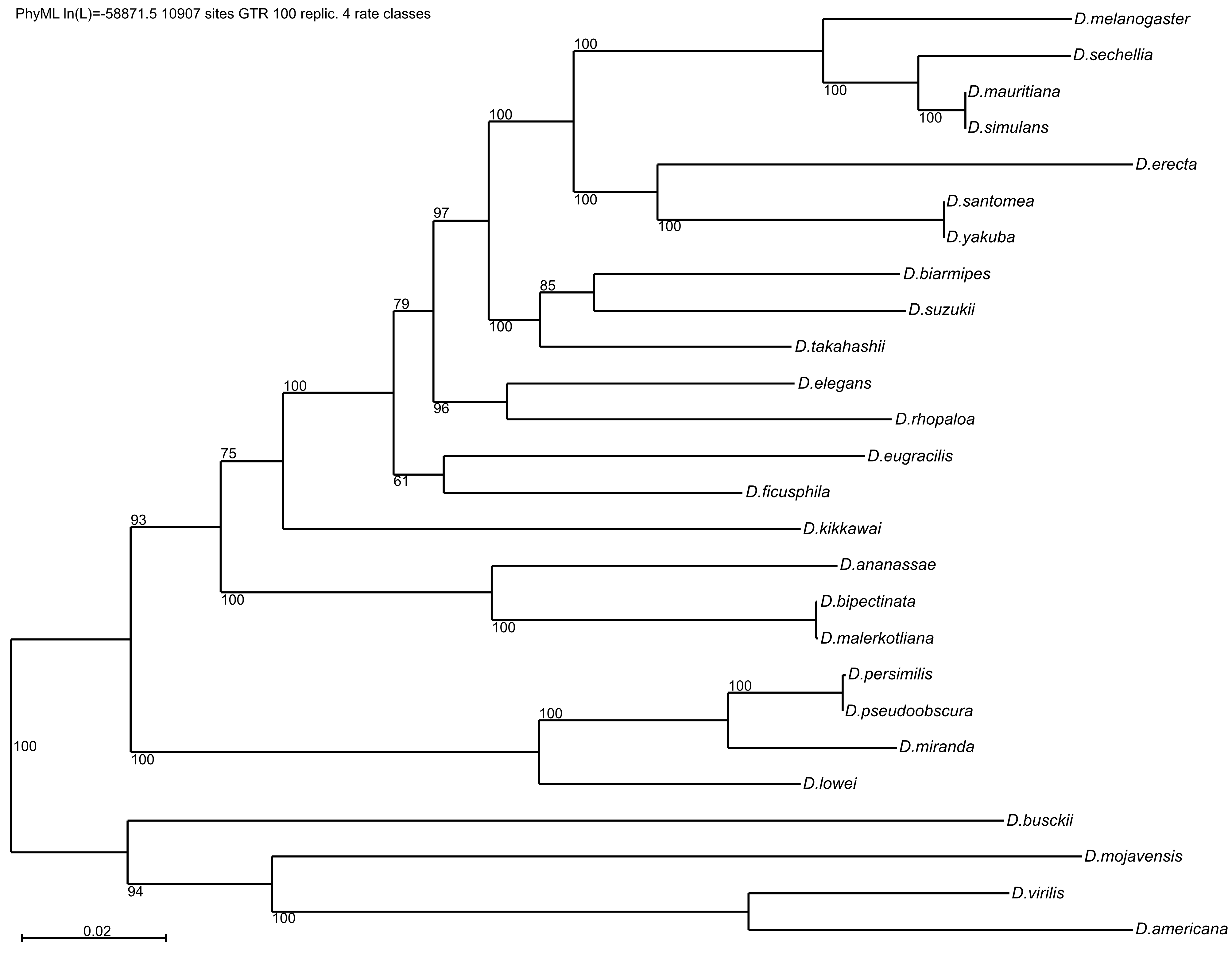

Supplement: Mitochondria phylogenetic tree [file rsbl20160407supp3.png]
